# The Impact of Two Elicitors and Harvest Ripening Stage on the Quality of Monastrell Grapes and Wines

**DOI:** 10.3390/biom15040474

**Published:** 2025-03-24

**Authors:** Rocío Gil-Muñoz, Maria José Giménez-Bañón, Juan Antonio Bleda-Sánchez, Juan Daniel Moreno-Olivares

**Affiliations:** Instituto Murciano de Desarrollo Agrario y Medioambiental, IMIDA, Ctra. La Alberca s/n, 30150 Murcia, Spain; mariaj.gimenez8@carm.es (M.J.G.-B.); juanantonio.bleda@carm.es (J.A.B.-S.); juand.moreno5@carm.es (J.D.M.-O.)

**Keywords:** methyl jasmonate, grape pomace extract, phenolic composition, maturation

## Abstract

In response to climate change, there is a decoupling between technological and phenolic maturity in vineyards. This study investigates the application of elicitors, specifically methyl jasmonate (MeJA) and grape pomace extract (GPE), administered at veraison and one week later over two consecutive seasons. Samples collected at 21 and 23 °Brix reveal delayed ripening for both treatments. MeJA significantly impacted extractable anthocyanins, the seed maturity index, and total anthocyanins, with optimal results at 23 °Brix compared to the control. GPE elevates proanthocyanin content at the same maturity level. Although the effects in wines are less pronounced, the MeJA-treated grapes harvested at 23 °Brix produce wines with reduced alcohol content, enhanced color intensity, and increased EGC-ext. Finally, a discriminant analysis indicated that the MeJA-treated grapes at 23 °Brix differed most significantly from the control, with seasonal variations playing a crucial role. Thus, MeJA 23 °Brix treatment demonstrated the most promising results, warranting further exploration with complementary winery technology to maximize its potential in vinification.

## 1. Introduction

Grapevine (*Vitis vinifera* L.) is a crop cultivated around the world and one of the most economically important fruit trees, mainly due to the wine industry [1], but since some time ago, it has been suffering from the impacts of climate change. This trend will continue to worsen in the next few decades regardless of the implementation of mitigation measures [2], particularly with more incidences occurring in arid or semi-arid areas such as southeastern Spain, where temperatures are high and rainfall is scarce. Different effects are occurring in this crop as a consequence of this new meteorological phenomenon, affecting the quality of grapes and wines.

On the other hand, wine quality is directly related to the berry chemical composition, which depends on various metabolites; the timing of ripening; and the synchrony of ripening of the skins, seeds, and pulp, which affect the type and style of wine that can be produced [1]. Therefore, harvest decisions remain a key management factor that allows a desired wine style to be obtained due to its marked effect on the phenolic and technological maturation of the grapes [3]. Despite the variability in style and taste, ripe fruits must include sufficient levels of sugar and secondary metabolites that contribute to the wine sensory profile (i.e., color, aromatics, flavor, and mouthfeel) [4]. One criterion is to reach the optimum sugar levels in these warmer seasons; however, they are not always concomitant with similar maturity in color, flavor, or aroma [5,6,7], culminating in the suggestion that ‘sugar ripeness’ is no longer coordinated with ‘phenolic’ ripeness [8,9], which affects the final quality of the wines made with them.

Different strategies can be used to increase the secondary metabolites in grapes to improve their quality and, at the same time, to try to decrease the uncoupling between technological and phenolic maturity. One of these strategies is the use of elicitors, which induce secondary metabolite accumulation as an important stress response that depends on biotic or abiotic elicitors as a regulatory signal that coordinates the expression of multiple biosynthetic genes [10]. The polyphenol content in wines depends on both their biosynthesis and accumulation in the berry during grape ripening and their extraction from the solid parts (seeds and skins) to the juice during the fermentation–maceration process [11,12,13]. According to some studies, there is a close relationship between high-quality wines and high polyphenolic concentration [12,14].

One of the most used elicitors in viticulture is methyl jasmonate (MeJA), which has been particularly reported in plants to potentially activate the synthesis of secondary metabolites afterward MeJA perception. Jasmonic acid (JA) and its volatile derivative methyl jasmonate (MeJA), known as jasmonates (JAs), are considered hormones acting in the regulation of a wide range of physiological processes in plants, including growth, photosynthesis, reproductive development, and responses to abiotic and biotic stresses [15,16]. The JA accumulated in tissues can be quickly converted into MeJA, a strong elicitor that acts as a signal molecule triggering a wide range of biosynthetic pathways and secondary metabolites [17,18,19]. Specifically, in wine grapes, several reports have shown that MeJA treatments in vineyards led to increasing phenolic contents, mainly anthocyanins, flavonols, and stilbenes, on grape and wine although huge differences between growing season and varieties were found [20,21,22,23].

On the other hand, pomace grapes are one of the most commonly used winemaking products, and annually, millions of tons of pomace grapes are produced as a byproduct, a revaluable resource with many potential uses. Currently, there is a growing interest in the usage of agricultural and horticultural residues in sustainable agriculture, such as fertilizers, biopesticides, and biostimulants. One of the most important bioactive principles that make pomace grape valuable as biostimulants is phenolic compounds treatment with grape pomace extract (GPE), which efficiently induces hypersensitive reaction-like lesions, with cell death evidenced by Evans Blue staining of tobacco leaves [24]. Phenol-rich fruit residues are known for their stimulative effects on crop plants. For instance, an experiment conducted on maize showed that fruit pomace hydrolysates resulted in significant increases in protein content and in various classes of phenolic compounds, although mixed results were obtained for sugars—sucrose, glucose, and fructose—with major decreases in the former [25]. Moreover, Gil-Muñoz et al. [26], in a study carried out in Monastrell, used different elicitors as a pomace grape extract and improved the phenolic composition in grapes and wines.

Numerous studies intend to improve the phenolic composition in grapes and wines to achieve a reduction in the decoupling between the different types of grape maturity and thus alleviate the consequences of climate change. Nevertheless, no information is available in these papers regarding the effects of the use of MeJA and GPE grape extract treatments on grapes harvested at two ripening moments. Only recently has a 10-day delay in the technological maturity (°Brix and pH) as a consequence of MeJA vineyard treatments been reported in the wine variety ‘Sangiovese’ [10] and Gómez-Plaza et al. [23] studied the application of BTH and MeJA on three different varieties during the ripening period. Under this framework, the main focus of this research is to provide an overview of the effect of two different elicitors (MeJA and GPE) on the phenolic composition of grapes and wines harvested at two different moments during two consecutive seasons to establish if the elicitors can contribute to diminishing the decoupling between phenolic and technological maturity.

## 2. Materials and Methods

### 2.1. Plant Material, Field Treatments, and Meteorological Information

This study was performed during two growing seasons (2022 and 2023) in Monastrell *Vitis vinifera* L.cv on Ritcher 110 rootstock, which were 10 years old and planted 2.5 × 3 m in an experimental field located in Cehegin (Murcia, Spain). Three treatments were carried out, each one in triplicate, at veraison and one week later, with 10 vines per replicate: (i) control (water), (ii) MeJA (10 mM), and (iii) grape pomace extract (GPE) (10 g/L) prepared as reported by Goupil et al. [24]. Briefly, pomace from a Monastrell vinification (after pressed) was freeze-dried and then extracted with ethanol (30%), and the extract was concentrated in a rotavapor (BUCHI Ibérica S.L.U, Barcelona, Spain) to eliminate all the ethanol used previously. Then, after its application to the plants, its pH was adjusted to 3.2.

The grapes were harvested at two ripening moments, when control grapes reached 2.1 ± 0.5 °Brix and 23 ± 0.5 °Brix, respectively, in order to consider the influence of treatment on the ripening process. Regarding meteorological information, total precipitation and average temperature during the ripening period (July–September) were 15.5 mm and 24.0 °C in 2022 and 18.5 mm and 24.2 °C in 2023 (data were recorded by the Agricultural Information System of the Region of Murcia (SIAM)).

### 2.2. Vinifications

The grapes were manually harvested into boxes and taken to our experimental winery, located in Jumilla. The winemaking process adhered to the traditional vinification protocol using 100 L stainless steel tanks. During the destemming and crushing of the grapes, they were treated with 50 mg/kg of potassium metabisulphite. Commercial yeast (Zymaflore FX10 *Saccharomyces cerevisiae*, 20 g/100 kg) was then added. Acidity was adjusted with tartaric acid until a value of 5.5 g/L was reached. Alcoholic fermentation was conducted at 25 °C, and temperature and tank density were monitored twice daily, accompanied by aerated pumping over. The maceration period lasted 14 days. The wine was subsequently drained and grape skins pressed using a pneumatic press. Finally, the wines were racked and cold-stabilized. Samples for the analyses were taken in triplicate at the end of alcoholic fermentation.

### 2.3. Physicochemical Parameters in Grapes and Wines

Different parameters were evaluated at the two chosen ripening moments (21 °Brix and 23 °Brix): total soluble solids, pH, total acidity, tartaric acid, and malic acid. Total soluble solids were measured using an Abbé-type refractometer (Atago RX-5000), and pH and total acidity using an automatic titrator (Metrohm, Herisau, Switzerland) with 0.1 N NaOH. Finally, tartaric acid and malic acid were assessed using a CETLAB 600 automatic analyzer (Microdom, Taverny, France).

### 2.4. Extractability Parameters in Grapes

The grapes’ phenolic potential was calculated according to the method described by Saint Cricq et al. [27], macerating the grapes for 4 h at two pH values (3.6 and 1.0). The anthocyanin contents of the two solutions (extractable anthocyanins and total anthocyanins) were then chemically assayed by measuring the absorbance of the samples at 520 nm at pH 3.6 and pH 1, while the total phenol content (TP) was calculated by measuring the absorbance of the solution at pH 3.6 at 280 nm. The original pH 3.2 solution was exchanged for one of pH 3.6, which is better suited to the must from the Jumilla localization. The phenolic potential was calculated as follows:

Index of Cellular Maturity (%) (IMC) = [ApH1 − ApH3.6)/TPpH3.6] × 100

Seed Maturity Index (%) (SMI) = [(TPpH3.6 − ((ApH3.6 × 40)/1000))/TPpH3.6] × 100

### 2.5. Spectrophotometric Parameters in Wines

Total wine anthocyanins were determined using the colorimetric method based on the Puissant–Léon technique with an automatic analyzer CETLAB 600 (Microdom, Taverny, France). Color intensity (CI) and total polyphenols (TPs) were determined using a Shimadzu UV/visible spectrophotometer 1600PC (Shimadzu Corporation, Kyoto, Japan). CI was calculated as the sum of the absorbance at 620 nm (blue), 520 nm (red), and 420 nm (yellow) in undiluted wine [28], and TP was analyzed by measuring the absorbance at 280 nm [29]. Finally, tannins were measured using the methyl cellulose precipitation methodology [30].

### 2.6. Analysis of Phenolic Compounds in Grapes and Wines

#### 2.6.1. Anthocyanins and Flavonols

The remaining 20 berries from each replicate were randomly selected from various clusters. The grapes were peeled using a scalpel, and the skins were stored at −20 °C until analysis. For the analysis, 2 g of skin samples was immersed in 40 mL of methanol in hermetically sealed tubes and placed on a stirring plate at 150 rpm and 25 °C. After four hours, the methanolic extracts (or in the case of wine, we took 2 mL) were filtered through 0.45 μm nylon filters (OlimPeak; Teknokroma, Barcelona, Spain) and analyzed by high-performance liquid chromatography (HPLC) following the method described by Gil-Muñoz et al. [31] As an external standard, anthocyanins were quantified at 520 nm using malvidin 3-O-glucoside chloride (Extrasynthèse, Genay, France). Flavonols were quantified at 360 nm using quercetin (Sigma–Aldrich, Madrid, Spain) as an external standard.

#### 2.6.2. Proanthocyanidins

Proanthocyanidins from skin grapes were analyzed using a previously describe method [32,33]. Briefly, skins from ten berries were separated, rinsed with water, and then extracted with acetone/water (2:1) for 24 h on an orbital shaker. The extract was concentrated to remove acetone, and the residue was redissolved in 2 mL of methanol. For wines, the samples were prepared by the method described by Pastor del Rio and Kennedy [34]. Wine samples (5 mL) were evaporated, re-dissolved in water, and passed through a C18-SPE column. Tannins were eluted with methanol, evaporated, and redissolved in methanol. Different cleavage products (terminal and extension units), mDP (mean degree polymerization), % galloylation (% gal), and total tannin content were calculated according to Gómez-Plaza et al. [23].

### 2.7. Statistical Analysis

Significant differences between treatments and season for each variable were assessed by analysis of variance (ANOVA) using Statgraphics Centurion Version 18.1.14 (B 2023 Statgraphics Technologies, Inc., The Plains, VA, USA). Least significant difference (LSD) tests were used to compare the means, and differences were considered statistically significant at *p* < 0.05. In addition, discriminant analyses were performed to determine whether the groups were sufficiently discriminated based on the original variables available.

## 3. Results and Discussion

### 3.1. Physicochemical Parameters in Grapes and Wines

As shown in Table 1, samples were harvested at two different ripening stages for both treated and control grapes. As is evident, this fact influenced the results obtained for the other measured parameters. Regarding maturity grade, although the grapes were harvested at 21 °Brix and 23 °Brix, we can observe how both treatments delayed the ripening process a little and therefore the amount of sugars accumulated in the berry. These results could indicate a possible solution for diminishing the uncoupling of technological and phenolic maturity in berries. Several authors such as Paladines-Quezada et al. [35] or Gil-Muñoz et al. [36] have also shown these same results, finding a delay in the ripening process of grapes after applying MeJA in Monastrell grapes, or Garde-Cerdán et al. [37] in Tempranillo grapes in one of the two years of study. Moreover, Gil-Muñoz et al. [38], in a study carried out in Monastrell during the ripening period, showed how the evolution of the accumulation of sugars in the berries was faster in the control grapes than in the rest of the grapes treated with MeJA in its different forms (conventional and nanoparticles form).

As expected, total acidity was higher at 21 °Brix than at 23 °Brix across the two factors studied: treatment and season. No differences were found among treatments, but with regard to year, the highest values were recorded in 2022 for both ripening stages, while the lowest value was observed in 2023 at 23 °Brix. Climatological conditions could have influenced these results, as the highest temperatures were reached during the second season in July and August compared to 2022 in the same months. However, other studies observed an increase in total acidity in Monastrell [31,36,39] and Tempranillo grapes [40] treated with MeJA.

Regarding pH, in general, the highest values were observed at 23 °Brix. Among the treatments, the highest values were found in the control and MeJA-treated grapes, while the lowest values were observed for the MeJA-treated grapes at 21 °Brix. Additionally, the lowest pH values were recorded in both years at 21 °Brix, while the highest values were observed in 2023 at 23 °Brix. Again, climatological conditions could have influenced these results, as the highest temperatures were reached during the second season. In this context, authors such as Mira de Orduña [41] explained that lower acidity levels are usually correlated with higher grape pH, though the relationship is affected by potassium accumulation, which itself depends on temperature. Other authors, such as Paladines-Quezada et al. [42] and Ruiz-Garcia et al. [39], also stated that the weather differences between years could have influenced the physicochemical composition of grapes treated with different elicitors.

Tartaric and malic acids are two of the main acids found in grapes. Tartaric acid values increased during the ripening period, with the highest values recorded at 23 °Brix for the two studied factors. Among the treatments, the lowest values were shown by GPE at 21 °Brix. Regarding season, tartaric acid values were higher in 2022 compared to 2023, although there were no statistical differences among the highest values obtained at 23 °Brix. Malic acid, on the other hand, decreased during the ripening period. The lowest values were recorded for the GPE at 23 °Brix, and considering season, the lowest value was observed in 2023 at 23 °Brix. According to these results, authors such as Huglin and Schneider [43] stated that while tartaric acid is relatively stable with regard to temperature effects, malic acid levels are tightly dependent on maturity and temperature, decreasing with higher temperatures, as in the second year studied.

Regarding the physicochemical parameters measured in wines at the end of alcoholic fermentation (Table 2), as is logical, the alcohol content was lower at 21 °Brix than at 23 °Brix, with statistical differences observed for all treatments at both harvest moments. The highest alcohol content was found in wines from the control and GPE-treated grapes at 23 °Brix, while the lowest value was observed in wines from the MeJA-treated grapes. These results can be explained by the fact that the harvest time was set at 21 °Brix; in fact, the treated grapes, especially those treated with MeJA, had slightly less alcohol content, which is justifiable by the slight delay in ripening. This fact is of great importance since this delay in technological maturity could lead to less alcoholic wines but of higher quality and more in line with the preferences of current consumers. Authors such as D’Onofrio et al. [10] observed in cv. Sangiovese that MeJA applied on the whole canopy led to a delay in berry ripening and consequently diminished alcohol content in wines. However, the season had little influence on the results, with the highest alcohol content recorded during the first year in wines from grapes harvested at 23 °Brix.

Since the alcoholic fermentation proceeded without any issues, the results for volatile acidity and reducing sugars were normal and complied with the required legislation. Volatile acidity was lower at 21 °Brix than at 23 °Brix for all studied factors. Among the treatments, there were no statistical differences; however, when considering the season, differences emerged, with the lowest value in the first season in wines from grapes harvested at 21 °Brix and the highest in 2023 in wines from grapes harvested at 23 °Brix. The same trend was observed for reducing sugars, with the highest values at 23 °Brix, though no statistical differences were found among treatments. However, differences were observed with higher values at 23 °Brix in both seasons.

The results for other parameters related to acidity levels varied. Total acidity was similar in wines from treated and untreated grapes at 21 °Brix but higher in control wines from 23 °Brix grapes and across the season at 23 °Brix. Regarding tartaric acid, statistical differences were found only between years, with the highest value in wines from grapes at 21 °Brix in both studied seasons. Malic acid was higher in control wines made from 21 °Brix grapes and lower in wines from GPE-treated grapes at 23 °Brix. This parameter also showed seasonal differences, with the highest value in wines from grapes harvested at 21 °Brix in both seasons. Finally, the highest pH values were found in wines from 21 °Brix grapes, except for wines from the MeJA-treated grapes at 23 °Brix and the 2023 vintage. This point is of great importance because increased pH values also favor oxidative reactions [44] and may affect wine color, taste, and aroma because it favors the formation of the colorless hemiketal anthocyanin form, reducing wine color in young red wines [45].

### 3.2. Extractability Parameters in Grapes

The extractability assay relates to the ease with which compounds of interest are released during the winemaking process. As shown in Table 3, the treatments affected the results obtained for the different parameters measured. For example, extractable polyphenols were higher in control grapes at 21 °Brix than in the other two treatments. This suggests that the application of elicitors in the field can lead to a thickening of the cell wall as a defense mechanism of the plant, which can affect the extractability of compounds of interest into wine during winemaking. Authors such as Apolinar-Valiente et al. [46] suggested that the composition of polysaccharides and oligosaccharides from the cell wall in Monastrell wines affected the release of compounds in wines from grapes treated with MeJA and other elicitors. Finally, the results varied by year, with the highest values found at 23 °Brix in 2022, while in 2023, the highest values of extractable polyphenols were observed at 21 °Brix.

Regarding extractable anthocyanins, in all treatments, the anthocyanin concentration was higher at 23 °Brix than at 21 °Brix, with the highest concentration observed in MeJA-treated grapes at 23 °Brix. This indicates a greater synthesis of anthocyanin compounds when treating the grape with this elicitor. Different studies have shown the effect of MeJA on anthocyanin content in grapes. For example, Portu et al. [47] observed a significant increase in berry total anthocyanin and total polyphenols after MeJA foliar application in Grenache. In addition, seasonal differences were found for this parameter, with the highest anthocyanin concentration observed during the first studied year, peaking in 2022 at 23 °Brix. Similar results were found when analyzing total anthocyanins (at pH 1), with the highest concentrations recorded at 23 °Brix, showing differences among treatments. The highest value of anthocyanins was found in the MeJA-treated grapes at 23 °Brix. This trend was consistent across the maturity grade factor, and as with extractable anthocyanins, concentrations were higher in 2022, with the highest values observed in 2022 at 23 °Brix.

Another important parameter in extractability is the IMC (index of cellular maturity), which expresses the ease of releasing anthocyanins during winemaking. As shown in Table 3, the values were generally lower at 23 °Brix, except in the control grapes, indicating that greater maturity leads to a higher release of phenolic compounds into the wine. The lowest value was obtained from the GPE-treated grapes, suggesting that a higher amount of phenolic compounds could be released during winemaking compared to the control or MeJA-treated grapes.

Regarding the studied seasons, this parameter had lower values in 2023 compared to 2022, with the lowest value obtained in 2023 in grapes harvested at 23 °Brix. The extractability of anthocyanins also increases during grape ripening as a consequence of cell wall degradation by pectolytic enzymes [48]. On the other hand, differences in polysaccharides based on galactose and arabinose, cellulose content, and the degree of pectin methylation may also be responsible for differences in anthocyanin extractability [49,50].

Finally, the SMI (seed maturity index) indicates the contribution of tannins from seeds to wine. As expected, the value of this parameter was higher at 21 °Brix than at 23 °Brix, indicating a greater amount of tannins in the seeds at this stage of grape development. Several authors have found that the highest synthesis of tannins occurs after fruit set, which happens a few weeks before veraison [51,52]. This is when the highest concentration of tannins is reached. From this point onwards, the skin proanthocyanidins either slightly decrease or remain stable [51]. Among the treatments, the lowest value was found in the MeJA-treated grapes at 23 °Brix, although all of them showed statistical differences between the two maturity grades. On the other hand, statistical differences were also found between seasons at the two maturity grades, with the highest value at 21 °Brix and the lowest result at 23 °Brix in both years. These results would again indicate the influence of vintage as a factor in this study.

### 3.3. Spectrophotometric Parameters in Wines

Different spectrophotometric parameters related to the phenolic composition and color were evaluated in the wines at the end of alcoholic fermentation (Table 4).

Regarding parameters related to phenolic composition, anthocyanins, tannins, and total phenols were analyzed. As can be seen, there were no statistical differences in total anthocyanins among the treatments, although it was observed that wine from GPE-treated grapes had the lowest anthocyanin quantities, while the MeJA-treated grapes at 23 °Brix showed the highest concentration of these compounds. These results are consistent with the extractability data shown for total and extractable anthocyanins (Table 3) and as explained previously, the effect of the elicitors on the cell wall could explain these results.

On the other hand, season only influenced the wines from grapes harvested at 21 °Brix, with the lowest levels of total anthocyanins observed in 2021 and the highest concentration of these compounds in 2023 at that maturity grade. As was the case with other measured parameters, climatological conditions could have influenced these results, as the highest temperatures were reached during the ripening period in 2023 compared to 2022, contributing to a higher biosynthesis of these compounds.

In general, lower values of tannins were observed in wines from treated and untreated grapes harvested at 21 °Brix. Regarding season, differences were observed between years, with higher results in 2023 compared to 2022. Specifically, the highest tannin content was found in wines from 2023 with 23 °Brix grapes and the lowest in wines from grapes harvested at 21 °Brix in 2022. Finally, total phenols (TPs) showed the highest values in wines from grapes harvested at 23 °Brix, with no differences among treatments or ripening degrees. The levels of this parameter were lower in 2023 compared to 2022, with the highest value in wines from 2022 made with grapes harvested at 23 °Brix and the lowest in wines from 2023 with grapes matured at 21 °Brix. Authors such as Paladines-Quezada et al. [42] showed an increase in TP in MeJA-treated wines from Merlot in one of the two seasons studied.

With respect to color parameters, total intensity, tint, and the CIElab parameters were analyzed. Total intensity (CI) did not show statistical differences among treatments for both maturity degrees, although it can be observed that the most intense wines were those from the MeJA-treated grapes harvested at 23 °Brix, and the lowest intensity was shown in wines from the GPE-treated grapes at any Brix level of origin. These results are consistent with the results obtained for anthocyanins in the different treatments, as CI and anthocyanins are closely related. Authors such as Ruiz-García et al. [38] and Paladines-Quezada et al. [42] also reported increases in CI in wines from Monastrell grapes treated with MeJA. Other authors, such as Portu et al. [20,22], also observed this increase in varieties such as Tempranillo and Grenache treated with MeJA.

The tint values ranged between 0.495 and 0.570, corresponding to wines from control grapes at 21 °Brix and 23 °Brix, respectively, thus showing the lowest and highest value obtained for this parameter. Regarding season, the highest values were found in 2023 compared to 2022, with the lowest taint value observed in 2022 in wines from grapes harvested at 21 °Brix and the highest in 2023 at 23 °Brix.

For red wines, CIELAB parameters help describe their appearance: L* (Lightness) indicates brightness, with darker reds having lower values; a shows the amount of red, usually higher in young wines; b* reflects yellow hues, typically lower in reds; H* (Hue) describes the tone, more purple in young reds and more brown in aged ones; and C* (Chroma) measures color intensity, with higher values indicating more vibrant colors. In our study, the lowest values were found in wines from grapes at 23 °Brix (MeJA-treated and untreated) according to the results obtained in CI for these wines, as well as in GPE grapes for parameters b* and H*. In general, the highest values were found in wines from GPE-treated grapes at 21 °Brix (L*, a*, C*) or at 23 °Brix (b*, H*), indicating less intense wines.

Finally, for the seasons, the CIElab parameters were lower in wines from 23 °Brix, except for H*, and the highest values were found in wines from 2021 at 21 °Brix. By contrast, authors such as Ruiz-García et al. [39] found a positive effect of MeJA in wines from grapes treated with MeJA, presenting higher CI and TP and a significantly lower L*, indicating darker wines.

### 3.4. Phenolic Composition in Grapes and Wines

#### 3.4.1. Anthocyanins

Anthocyanins are the main compounds responsible for the color grapes and wines and their accumulation starts at veraison and reaches a maximum at harvest time [52,53]. The anthocyanin content of grapes is shown in Table 5, and the concentration of these compounds, as expected, was influenced by the degree of maturity in grapes. For all the factors studied—treatment and vintage—we consistently obtained higher concentrations of anthocyanins at 23 °Brix. Gómez-Plaza et al. [23] reported that anthocyanin starts to accumulate in grape skins at veraison and reach their peak around harvest time, similar to the result obtained by González-Lázaro et al. [54].

Total anthocyanins did not show differences among treatments at 21 °Brix; however, at 23 °Brix, the highest total anthocyanin content was observed in MeJA-treated grapes, and the lowest content in GPE-treated grapes. Ruiz-García et al. [55] reported that the application of BTH+MeJA doubled the quantities of grape anthocyanins in ‘Monastrell’ grapes. In other studies, Ruiz-García et al. [39] also found increased anthocyanin content during two consecutive years when MeJA was applied to ‘Monastrell’ grapes. Another study carried out by Giménez-Bañón et al. [56], in which Monastrell grapes were treated with MeJA and nanoparticles with MeJA, showed an increase with both treatments, although this was more notable when MeJA was used conventionally. Other authors have observed these increases in other varieties such as Tempranillo [20,21], Syrah [54], or Graciano [22]. However, Gómez-Plaza et al. [23], in a ripening study on Monastrell grapes treated with different elicitors, observed higher anthocyanin levels in the treated grapes at the fourth sampling date, although differences were maintained until harvest. The differences between treatments were more evident in the middle of the ripening period compared to the control grapes. Finally, in line with our results, Gil-Muñoz et al. [26] did not find any increase in the content of anthocyanins in grapes treated with GPE compared to control grapes.

Individual anthocyanins were analyzed by liquid chromatography, and as shown in Table 5, differences among treatments were found. Five anthocyanin glucosides together with acylated derivates from two of them (Pn-3g and Mv-3g) were detected. Mv-3g was the anthocyanin present with the highest concentration, as is common in *Vitis vinifera* varieties.

With respect to treatments, the highest concentrations of Df-3g, Pet-3g, Mv-3g, and Mv-ac-3g were observed in MeJA-treated grapes harvested at 23 °Brix. The highest results for Cy-3g, Pn-3g, Pn-ac-3g, and Mv-ac 3g were seen in the control and MeJA-treated grapes at 23 °Brix. Generally, the lowest content of these compounds at 23 °Brix was found in the GPE-treated grapes. Authors such as Gil-Muñoz et al. [36] also observed a higher increase in the different individual anthocyanins when Monastrell grapes were treated with MeJA. On the other hand, González-Lázaro et al. [54] reported an increase in Cy-3g and Pn-3g in MeJA-treated grapes from Tempranillo. However, when the treatment was combined with urea, the authors observed an increase in Df-3g, Cy-3g, Pet-3g, Pn-3g, Mv-3g, Dp-cum3g, Pet-cum3g, and Mv-cum3g compared to the control grapes. As with the case when we talked about total anthocyanins, no statistical differences were found between the treatments for grapes harvested at 21 °Brix. Therefore, it could be concluded that it is necessary for the grapes to have a certain degree of ripening so that the effect of the elicitors is reflected in a greater biosynthesis of anthocyanins, as Gómez-Plaza et al. [23] showed in a study carried out in three different grapes varieties during the ripening period.

Season influenced the results obtained for individual anthocyanins, with the highest concentrations observed in 2022 compared to 2023, although only a few statistical differences were noted. These results would be in agreement with those obtained in the extractable anthocyanins calculated in Section 3.2 of this article. Authors such as Portu et al. [22], in a study on MeJA-treated Tempranillo grapes over two consecutive years, observed that the treatment had a greater influence on non-acylated anthocyanins in 2015, while the effect was greater on acylated forms in 2016. However, other studies demonstrated that in Monastrell, MeJA treatment had a greater influence on acylated derivatives than on non-acylated compounds. Paladines-Quezada et al. [35], in a study on Monastrell grapes treated with MeJA, BTH, and MeJA+BTH during two ripening periods, observed a higher anthocyanin increase in grapes treated with MeJA+BTH at veraison or mid-ripening compared to any other treatment. Finally, Gil-Muñoz et al. [38], in a study on Monastrell with different forms of MeJA application, revealed that acylated, acetates, and coumarate anthocyanins increased with MeJA treatments in all cases.

With respect to the results obtained for anthocyanins in wines (Table 6), the differences found were much smaller than those in grapes. These results could be explained by the fact that the application of elicitors could have produced changes in the structure and composition of the skin cell wall, leading to reinforcement of the skin cell wall, thus hindering full anthocyanin extraction during the winemaking process [42]. This fact could be corrected with the application of some technology in the winery that more exhaustively destroys the cell walls of the skin, releasing possible compounds of interest during the winemaking process.

No differences were found in total anthocyanins in the wines among treatments harvested at 21 °Brix or 23 °Brix, contrary to what was found in grapes. With respect to the individual compounds analyzed, we only observed higher concentrations of Pn-3g and Pn-3g pyr in wines from the control and MeJA-treated grapes harvested at 23 °Brix, and of Df-3g and Mv-ac-3g in wines from control grapes harvested at 21 °Brix. Additionally, acetyl vitisin A was higher in the control wines at 23 °Brix, and Mv-6 cum3g was higher in the MeJA wines at 21 °Brix. Authors such as Portu et al. [40] also noticed the absence of differences between control and MeJA wines regarding anthocyanins compounds. However, Gil-Muñoz et al. [36] showed an increase in wines from Monastrell, but not in Merlot from MeJA-treated grapes.

Finally, season was the most influential factor in the results obtained for these parameters. The highest total anthocyanin content was observed in the second vintage compared to the first, although no statistical differences were found between maturity degrees. With respect to the individual compounds, the highest concentrations of acetates and coumarates were found in 2023 at 21 °Brix and 23 °Brix, while monoglucosides showed different trends. For example, the highest content of Pt-3g and Mv-3g was observed in wines from grapes at 21 °Brix in 2021, and the highest Pn-3g content was found in wines from 2021 and 2023 when the grapes were harvested at 23 °Brix. Gil-Muñoz et al. [36], in a study carried out with three varieties (Syrah, Merlot and Monastrell) treated with MeJA and BTH, observed how the results were dependent on the variety. However, in the Monastrell variety, the effect was stronger than that in BTH, since OH-forms did not increase when the grapes were treated with BTH, although in wines, both treatments produced higher levels of glucosides and acetylated anthocyanins compared to the control samples, but not higher levels of coumarate anthocyanins. On the other hand, Paladines-Quezada et al. [35] observed how wines made in the 2016 season showed a higher concentration of total anthocyanins than those of 2017, but no differences were found between the treatments and control wines. Finally, Gil-Muñoz et al. [26] found a higher anthocyanin concentration when cold maceration was used in wines from grapes treated with BTH and GPE.

#### 3.4.2. Flavonols

Flavonols are yellow pigments implicated in the color attributes of white and red wines and have great importance due to their antioxidant and UV-screening capacity. For these reasons, it is one of the most studied classes of polyphenols.

With regard to flavonols, we identified 10 different glucoside, galactoside, and glucoronide compounds, such as myricetin, quercetin, laricitrin, kampherol, isorhamnetin, and syrigetin. With respect to flavonols in grapes (Table 7), the treatments did not have any effect on the total flavonols. As we can observe, the highest flavonol content was obtained in the control grapes matured at 23 °Brix. Although the MeJA- and GPE-treated grapes also showed higher quantities of flavonols in more mature grapes, only the control grapes showed statistical differences between the two harvest moments. Authors like Gil-Muñoz et al. [26] also found no differences between Monastrell grapes treated with MeJA and GPE compared to control grapes. However, other authors, such as Ruiz-Garcia et al. [55] showed an increase in flavonol content of MeJA-treated grapes at harvest. These same authors reported that years with higher humidity and lower temperatures may provide suitable conditions for pathogen development and consequently help elicitor-treated plants react more efficiently. On the other hand, Gómez-Plaza et al. [23], in a ripening study with Monastrell grapes, observed how the MeJA treatment only increased the flavonol concentration during the first few days, whereas during the last 3 weeks before harvest, the flavonol concentration between the MeJA-treated and control grapes did not differ. Portu et al. [21] also found no effect after the application of three different elicitors in Tempranillo grapes. Finally, contrary to our results, Gil-Muñoz et al. [38] did observe an increase in flavonol composition in Monastrell grapes treated with different MeJA treatments (conventional and nanoparticles).

Regarding seasons, we could observe that in 2022, the highest amount of flavonols was obtained, especially at 23 °Brix, showing statistical differences between the two maturity moments. However, in 2023, no statistical differences were found between those moments. Ruiz-García et al. [39] evidenced a more significant effect of treatments in 2010 on flavonol content, especially in Monastrell grapes treated with MeJA than in 2009, and Portu et al. [40] found significant differences between the control and MeJA-treated grapes from Tempranillo, especially in 2016 rather than in 2015. Finally, Ruiz-García et al. [55] observed in a study carried out on different Monastrell clones treated with MeJA that some clones increased their levels of flavonols while others decreased them. Once again, vintage was an essential factor in the results obtained.

In contrast, the individual flavonols analyzed showed different behaviors depending on the treatments applied. For example, Myr-3gU obtained the highest concentration in the MeJA-treated grapes at 23 °Brix, while Myr-3g and Quer-3g obtained the highest concentrations in the control and MeJA-treated grapes at 23 °Brix. For other compounds, such as Lar-3g and Syr-3g, the highest contents were found at 23 °Brix for the control and both treatments. In general, the highest flavonol concentrations were observed in grapes ripened at 23 °Brix, except for Quer-3gU. In this context, Portu et al. [40] observed an increase in Quer-3gU, Quer-3g, Kamp-3gal, and Kamp-3g in the MeJA-treated grapes from Tempranillo and Graciano in one of the studied years. On the other hand, Gil-Muñoz et al. [36] observed few differences in Monastrell grapes at harvest, with only Myr-3g showing a higher concentration in the MeJA-treated grapes than in control grapes and Iso-3g showing a higher concentration for the MeJA and BTH treatments.

Season also influenced the behavior of the individual compounds analyzed. The highest contents of Quer-3gal, Quer-3g, and Kam-3g were obtained in grapes matured at 23 °Brix in 2022. In 2023, the highest contents of Myr-3g and Myr-3gal were found in grapes harvested at 23°Brix. However, for Quer-3gU, the highest concentration was found in grapes matured at 21°Brix during the same year. Again, differences in climatological conditions between years could explain the variations in results, as these conditions can affect the biosynthesis of each individual compound differently.

In wines, contrary to what happened with anthocyanins, the flavonol results were more pronounced than in grapes, with significant differences found between treatments and season. In general, the highest flavonol content was observed in wines from grapes matured at 23 °Brix. Regarding treatments, the effect of MeJA was more noticeable in wines than in grapes, although the total flavonol content was similar to that obtained in control wines. Authors like Gil-Muñoz et al. [26] observed a highest flavonol concentration in wines elaborated using cold maceration with Monastrell grapes treated with BTH and GPE but not with MeJA.

For the individual compounds analyzed, the highest concentrations of Iso-3g were found in the control wines from grapes harvested at 23 °Brix. However, in wines from the MeJA-treated grapes matured at 23 °Brix, the highest amounts of Myr-3g and Lar-3g were observed. Finally, the control and treated wines showed similar results for Myr-3gal and Syr-3g. Authors such as Gil-Muñoz et al. [36] showed an increase in Myr-3g, Quer-3g and Quer-3gU in Merlot wines from MeJA-treated grapes, although this increase was not observed in Monastrell wines. Regarding the influence of season, the highest concentration of flavonols was found in the second year, in wines from grapes ripened at 23 °Brix. However, the highest content of Quer-3gal was observed in wines from grapes harvested at 23 °Brix in 2022, and the highest content of Kam-3g was found in wines from grapes harvested at 21 °Brix in the same year (Table 8).

#### 3.4.3. Proanthocyanidins

Proanthocyanidins (PAs) or commonly called “condensed tannins” are considered very important for grape and wine quality, since they participate in such important attributes as the astringency perception, bitterness, or color stability of wines [38].

The results corresponding to PAs in the skin and different parameter-related measures are shown in Table 9. As can be observed, the highest concentration in total PAs expressed as mg/kg grape was found in the control and GPE-treated grapes harvested at 23 °Brix. However, these results are not consistent with those found by Gil-Muñoz et al. [26], who did no show an increase in total PAs in Monastrell grapes treated with MeJA or GPE. If we take into account the results by µg/g berry, the control and MeJA treated grapes harvested at 21 °Brix expressed the highest quantities of these compounds; however, the lowest values were found in the control grapes harvested at 23 °Brix. Finally, when expressing total PAs content as µg/g skin, the control and treated grapes harvested at 21 °Brix obtained the highest concentration, while the opposite occurred at 23 °Brix. However, different results were obtained in studies conducted by various authors. For example, Gil-Muñoz et al. [57], in a study on two varieties (Monastrell and Tempranillo) treated with MeJA during two consecutive seasons, observed an increase in total PAs in grapes and wines for both varieties in only one year. In contrast, Gil-Muñoz et al. [38] showed the lowest concentration of PAs in grapes treated with MeJA in a conventional way during the ripening period; however, when MeJA was applied as nanoparticles, the PA level was higher. Finally, Gómez-Plaza et al. 233] found that MeJA-treated grapes obtained a higher total concentration of PAs in the skin than control grapes during the ripening period. Therefore, it is evident that the results depend on factors such as variety, season, or application form.

Other parameters related to PAs were measured as mDP, with the highest value found in the control grapes at 21 °Brix and the MeJA-treated grapes at 23 °Brix, while the lowest value was observed in the GPE-treated grapes at 23 °Brix. Gil-Muñoz et al. [38], in a study on Monastrell grapes treated with MeJA in conventional form and two different nanoparticles, did not show differences between the treated and untreated grapes at harvest time. However, other authors such as Ruiz-García et al. [39] and Gil-Muñoz et al. [36] observed an increase in mDP in Monastrell grapes treated with MeJA. With respect to % Gal, the MeJA-treated grapes obtained the highest percentages of galloylation. However, Gil-Muñoz et al. [38] and Ruiz-García et al. [39] did not find differences regarding % Gal between the MeJA-treated and untreated Monastrell grapes. Both parameters will influence the organoleptic characteristics of the wines, since a higher mDP is associated with a greater amount of tannins that come from the skins that give rise to softer tannins; however, a higher % Gal is associated with seed tannins that will give more astringent notes.

With regard to the composition of PAs, for skin terminal units, the highest values were reached by Cat-T, followed by Epi-T, and finally ECG-T. With respect to treatments, the highest concentrations of Cat-T and ECG-T were obtained with MeJA at 21 °Brix, while for Epi-T, the highest concentration was observed with MeJA at 23 °Brix. Regarding the season, the highest concentration of Cat-T was obtained in 2023 at 21 °Brix, and for ECG-T, it was highest in 2022 at 23 °Brix. Epi-T did not show differences between years and maturity stages. Gil-Muñoz et al. [57] found practically no differences between the treated and untreated grapes for Monastrell and Tempranillo for these measured parameters. Regarding extension units, the main constituent was Epi-Ext, with the highest concentration observed in the control grapes at 21 °Brix. No significant differences were found between treatments. For Cat-Ext, higher levels were observed in both treatments at 21 °Brix. Finally, EGC-Ext showed the highest concentrations in MeJA-treated grapes at both maturity stages.

The results of total PAs and the different related parameters in wines (Table 10) were less evident than those found in the grapes, as with anthocyanins. Although the highest concentrations of total PAs were shown in the wines from both treated grapes (MeJA and GPE), no statistical differences were observed with the control wines. Again, the effect that the treatments could have had on the cell wall could have conditioned the extractability of the compounds of interest during the winemaking process. Gil-Muñoz et al. [26] observed that wines elaborated in a traditional way from grapes treated with MeJA, the mixture of BTH+MeJA, and GPE obtained the highest concentrations of PAs; however, when cold maceration was used, higher results were only found in wines from the GPE-treated grapes. On the other hand, vintage as a factor showed the highest PA content in wines produced in 2023 with grapes harvested at 21 °Brix.

With respect to mDP or % Gal, no differences were found between treatments and only mDP in the second season obtained higher values at the two mature stages in comparison with 2022. However, for % Gal, the opposite occurred, showing the highest values in the first season. The percentage of tannin galloylation affects both the bitterness and astringency of wines [58].

Regarding the terminal units’ composition of PAs, a higher concentration of Epi-T was observed in the treatments and the control wines from grapes harvested at 23 °Brix, and a higher concentration of ECG-T was found in wines from GPE-treated grapes. In 2022, the highest concentrations of Cat-T, Epi-T, and ECG-T were obtained at 23 °Brix, while for Cat-T, the highest concentration in 2023 was at 21 °Brix.

With respect to the extension units, the treatments only affected the wines from MeJA-treated grapes, which obtained the highest concentration of EGC-Ext at both maturity stages. The same results were observed for EGT-Ext content. Mercurio et al. [59] reported that wines with a higher tannin content and a higher percentage of epigallocatechin, indicative of higher skin-derived tannin content, were better appreciated. Finally, the highest concentration of extension units was observed during the second season at 21°Brix.

### 3.5. Multivariable Discriminant Analysis

Discriminant analyses were used to check if we could classify our samples (grapes and wines) based on the two factors analyzed, treatment and year.

#### 3.5.1. Grape

**Figure 1:**
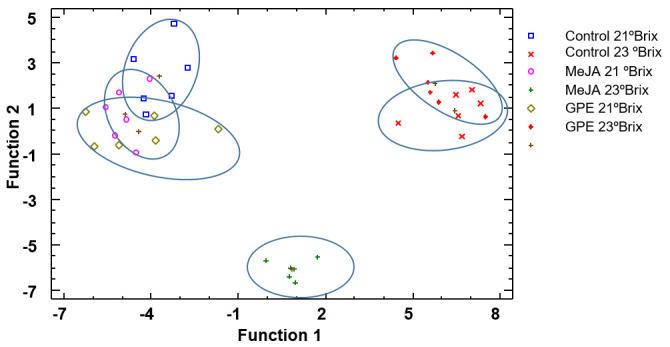
Discriminant analysis of grapes according to treatment. Abbreviations: MeJA 21 °Brix, grapes treated with methyl jasmonate and harvested at 21 °Brix; MeJA 23 °Brix, grapes treated with methyl jasmonate and harvested at 23 °Brix; GPE 21 °Brix, grapes treated with grape pomace extract and harvested at 21 °Brix; GPE 23 °Brix, grapes treated with grape pomace extract and harvested at 23 °Brix.

Figure 1 corresponds to the discriminant analysis using treatment as the discriminant factor. Three discriminant functions were calculated with a *p*-value lower than 0.05 and therefore statistically significant and allowed us to correctly classify 90% of the samples. The relative percentages for function 1 and function 2 were 65.8% and 23.8%, respectively. As can be observed, the separation of the treatments regarding the control samples was better at 23 °Brix than at 21 °Brix. Three different groups can be observed, as follows: the first, located on the left part of the figure, is all treatments harvested at 21 °Brix; the middle of the graphic is the group corresponding to MeJA-23 °Brix, the most different group in comparison with the rest of treatments; and finally, the control grapes and GPE-treated grapes harvested at 23 °Brix are located on the right side of Figure 1.

These results are logical since, for the parameters studied, the treatment that stood out the most was MeJA when the grapes were harvested at 23 °Brix. The standardized coefficients of the functions were used to discriminate between treatments, with the following variables having the highest discriminatory power: for function 1, total acidity, tartaric acid, and total anthocyanins, and for function 2, total acidity, pH, IMC, SMI, and total anthocyanins.

**Figure 2:**
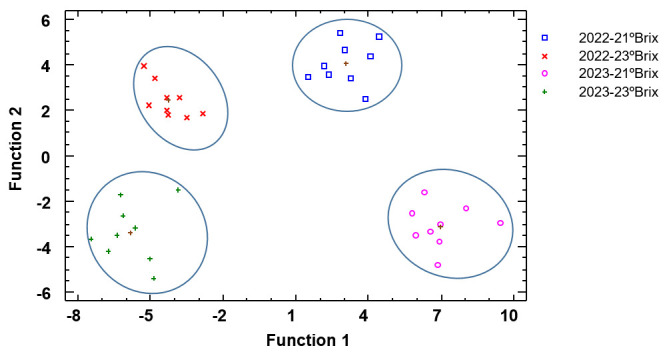
Discriminant analysis of grapes according to year. Abbreviations: 2022-21 °Brix, grapes harvested at 21 °Brix during the 2022 season; 2022-23 °Brix, grapes harvested at 23 °Brix during the 2022 season; 2023-21 °Brix, grapes harvested at 21 °Brix during the 2023 season; 2023-23 °Brix, grapes harvested at 23 °Brix during the 2023 season.

Figure 2 corresponds to the discriminant analysis using season as the discriminant factor. Three discriminant functions were calculated with a *p*-value lower than 0.05 and therefore statistically significant and allowed us to correctly classify 100% of the samples. The relative percentages for function 1 and function 2 were 67.7% and 26.7%, respectively. Clearly, the grapes harvested during the first season at 21 °Brix and 23 °Brix are situated in the positive part of function 2. In the same way, the grapes harvested during 2023 are located in the negative part of function 2. It can be observed that the grapes harvested in 2022 are closer than those harvested in 2023, indicating less differences between them than in 2023. With regard to standardized coefficients, for function 1, the variables with the highest discriminatory power were again total acidity, tartaric acid, and total anthocyanin and flavonols, and for fuction 2, total acidity, pH, total anthocyanin, and IMC.

#### 3.5.2. Wine

**Figure 3:**
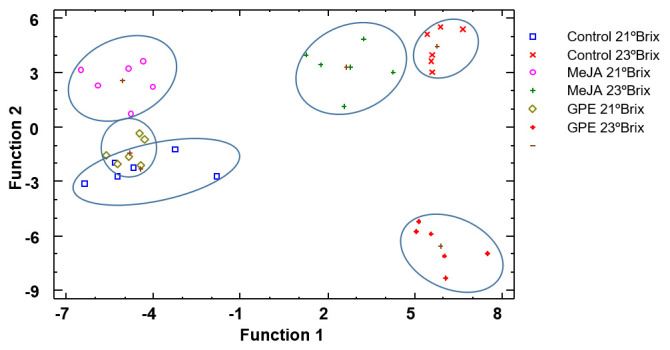
Discriminant analysis of wines according to treatment. Abbreviations: MeJA 21 °Brix, wines from grapes treated with methyl jasmonate and harvested at 21 °Brix; MeJA 23 °Brix, wines from grapes treated with methyl jasmonate and harvested at 23 °Brix; GPE 21 °Brix, wines from grapes treated with grape pomace extract and harvested at 21 °Brix; GPE 23 °Brix, wines from grapes treated with grape pomace extract and harvested at 23 °Brix.

Regarding wines, Figure 3 corresponds to the discriminant analysis using treatment as the discriminant factor. Three discriminant functions were calculated with a *p*-value lower than 0.05 and therefore statistically significant and allowed us to correctly classify 96% of the samples. As we can observe, just as with grapes, wines from grapes treated and harvested at 23 °Brix are better separated in the graph than those harvested at 21 °Brix. On the other hand, wines from treated grapes harvested during 2022 are situated on the left of Figure 3 or on the negative side of function 1. However, treated grapes harvested in 2023 are situated on the left side of the graphic or on the positive side of function 1; in addition, wines from MeJA-treated grapes and control grapes are closer than GPE-treated grapes. Finally, the wine from the grapes treated with GPE and harvested at 23 °Brix turns out to be further away and therefore more different. With regard to standardized coefficients, for function 1, the variables with the highest discriminatory power were again total acidity, tartaric acid, and total anthocyanin and flavonols, and for function 2, total acidity, pH, total anthocyanin, and IMC.

**Figure 4:**
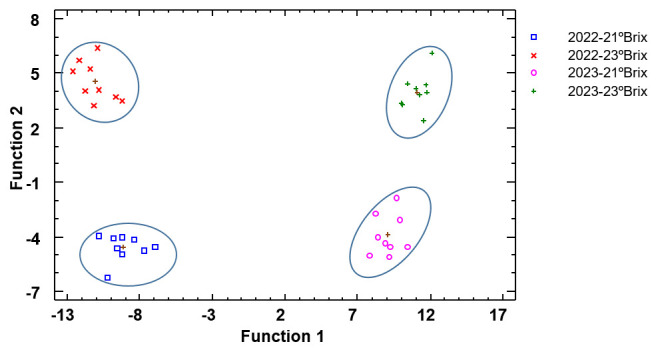
Discriminant analysis of wines according to season. Abbreviations: 2022-21 °Brix, wines from grapes harvested at 21 °Brix during the 2022 season; 2022-23 °Brix, wines from grapes harvested at 23 °Brix during the 2022 season; 2023-21 °Brix, wines from grapes harvested at 21 °Brix during the 2023 season; 2023-23 °Brix, wines from grapes harvested at 23 °Brix during the 2023 season.

Figure 4 corresponds to the discriminant analysis using seasons as the discriminant factor. Three discriminant functions were calculated with a *p*-value lower than 0.05 and therefore statistically significant and allowed us to correctly classify 100% of the samples. As in the grapes, a clear differentiation between years could be observed on the graph. Again, wines from grapes harvested in 2022 are situated on the left side of the graphic, and wines from grapes harvested in 2023 are located on the right side of Figure 4. The separation by vintage and time of harvest was clear. On this occasion, it seems that the wines from 2023 at the two stages of maturation are a little closer than those from 2022, indicating greater similarity between them. With regard to standardized coefficients, for function 1, the variables with the highest discriminatory power were IC, TP, taint, and total tannins, and for function 2, IC and total anthocyanins.

## 4. Conclusions

This study evaluated the effects of two elicitors, MeJA and GPE, on Monastrell vineyards to address the decoupling between phenolic and technological maturity. Grapes were harvested at 21 and 23 °Brix over two consecutive years. The measured parameters were influenced mainly by the season, although the treatments also showed an effect.

A delay in ripening was observed in the treated grapes overall when harvested at 23 °Brix compared to in the control grapes. These results influenced the rest of the measured parameters. The MeJA-treated grapes showed the highest anthocyanin concentrations, while the GPE-treated grapes showed the lowest, possibly due to cell wall thickening caused by its application affecting extractability parameters. In general, the MeJA-treated grapes at 23 °Brix showed higher extractable anthocyanins, and the highest concentration in total PAs expressed as mg/kg grape was found in the GPE-treated grapes harvested at 23 °Brix.

In wines, the results were less evident than in grapes, but wines from MeJA-treated grapes at 23 °Brix resulted in the lowest alcohol content and highest color intensity. The phenolic composition results were also less noticeable in the wines than in the grapes. MeJA-treated grapes at 23 °Brix showed the highest anthocyanin concentrations, while wines from GPE-treated grapes had the lowest at the same ripening stage. The highest flavonol content was observed in wines from grapes matured at 23 °Brix. MeJA’s effect was more noticeable in wines, although the total flavonol content was similar to that of the control wines. Regarding PAs, no differences were found among treatments for total PAs, mDP, or % Gal. However, with respect to the season, in the second year, mDP had higher values at both maturity stages compared to that in 2022. Wines from the GPE-treated grapes had the highest concentration, while MeJA-treated wines had the highest EGC-Ext concentration.

In summary, MeJA at 23 °Brix gave better results than GPE, suggesting that it could mitigate the decoupling of ripening in grapes, though further studies are needed to optimize its use.

## Figures and Tables

**Table 1:** Physicochemical parameters in grapes at harvest time.

	°Brix		Total Acidity *		pH		Tartaric Acid (g/L)		Malic Acid (g/L)	
	21 °Brix	23 °Brix	21 °Brix	23 °Brix	21 °Brix	23 °Brix	21 °Brix	23 °Brix	21 °Brix	23 °Brix
**Treatment**										
Control	21.6 b,α	23.32 d,β	3.76 b,α	3.28 a,β	3.90 b,α	4.08 d,β	4.30 b,α	5.13 c,β	2.58 c,α	2.14 b,β
MeJA	20.23 a,α	22.43 c,β	4.00 b,α	3.41 a,β	3.83 a,α	4.07 d,β	4.16 ab,α	4.84 c,β	2.58 c,α	2.19 b,β
GPE	20.38 a,α	22.82 cd,β	3.77 b,α	3.26 a,β	3.88 b,α	3.98 c,β	3.82 a,α	4.94, c,β	2.73 c,α	1.85 a,β
**Year**										
2022	21.70 b,α	22.82 c,β	3.77 b,α	3.79 b,α	3.89 a,α	3.98 b,β	4.87 b,α	5.00 b,α	2.45 b,α	2.48 b,α
2023	20.62 a,α	22.52 bc,β	3.54 ab,α	3.29 a,β	3.87 a,α	4.05 c,β	3.79 a,α	4.86 b,β	2.57 b,α	2.0 a,β

* Expressed as g/L tartaric acid. Abbreviations: MeJA: methyl jasmonate; GPE: grape pomace extract. Different letters in the same column (a–d) indicate significant differences according to the LSD test (*p* < 0.05) for the two studied factors (treatment and year) separately. For each quality grape parameter, different letters in the same row (α, β) indicate significant differences between harvest time (*p* < 0.05).

**Table 2:** Physicochemical parameters in wines at the end of alcoholic fermentation.

	Alcohol (%*v*/*v*)		Volatil Acidity (g/L)		Reductor Sugars (g/L)		Total Acidity (g/L)		pH		Tartaric Acid (g/L)		Malic Acid (g/L)	
	21 °Brix	23 °Brix	21 °Brix	23 °Brix	21 °Brix	23 °Brix	21 °Brix	23 °Brix	21 °Brix	23 °Brix	21 °Brix	23 °Brix	21 °Brix	23 °Brix
**Treament**														
Control	12.09 b,α	13.99 d,β	0.32 a,α	0.41 c,β	0.97 a,α	1.15 b,β	6.45 ab,α	7.20 d,β	3.57 b,α	3.51 a,β	2.82 a,α	2.57 a,α	2.36 c,α	1.90 abc,α
MeJA	11.33 a,α	13.19 c,β	0.37 abc,α	0.41 c,α	0.94 a,α	1.12 b,β	6.45 ab,α	6.65 c,β	3.56 b,α	3.57 b,α	2.85 a,α	2.51 a,α	1.80 ab,α	2.05 abc,α
GPE	11.78 b,α	13.80 d,β	0.34 ab,α	0.40 bc,α	0.94 a,α	1.10 b,β	6.31 a,α	6.64 bc,β	3.60 b,α	3.48 a,β	2.88 a,α	2.78 a,α	2.22 bc,α	1.66 a,β
**Year**														
2022	11.77 a,α	13.80 c,β	0.31 a,α	0.37 b,β	0.94 a,α	1.15 c,β	6.48 b,α	6.84 c,β	3.51 a,β	3.51 a,β	3.14 d,α	2.95 c,β	1.86 ab,α	1.75 a,α
2023	11.67 a,α	13.38 b,β	0.40 b,α	0.47 c,β	0.97 a,α	1.07 b,β	6.24 a,α	6.81 c,β	3.54 b,β	3.54 b,β	2.27 b,α	1.95 a,β	2.65 c,α	2.12 b,β

Abbreviations: MeJA: methyl jasmonate; GPE: grape pomace extract. Different letters in the same column (a–d) indicate significant differences according to the LSD test (*p* < 0.05) for the two studied factors (treatment and year) separately. For each quality wine parameter, different letters in the same row (α, β) indicate significant differences between harvest time (*p* < 0.05).

**Table 3:** Extractability parameters in grapes at harvest time.

	Extractable Polyphenols (mg/Kg)		Extractable Anthocyanins (mg/Kg)		Total Anthocyanins (mg/Kg)		IMC		SMI	
	21 °Brix	23 °Brix	21 °Brix	23 °Brix	21 °Brix	23 °Brix	21 °Brix	23 °Brix	21 °Brix	23 °Brix
**Treatment**										
Control	50.56 c,α	47.49 bc,α	335.81 a,α	481.1 b,β	577.09 a,α	876.33 c,β	40.78 ab,α	44.78 abc,α	73.29 c,α	59.31 b,β
MeJA	45.50 a,α	46.32 abc,α	319.65 a,α	538.37 c,β	667.44 ab,α	1005.57 d,β	51.91 c,α	45.31 abc,α	72.13 c,α	58.23 a,β
GPE	42.61 a,α	44.20 ab,α	305.42 a,α	444.1 b,β	568.35 a,α	735.5 b,β	46.35 bc,α	38.00 a,β	71.74 c,α	58.94 b,β
**Year**										
2022	47.91 bc,α	49.93 c,α	359.22 b,α	513.18 d,β	702.22 b,α	959.53 d,β	48.32 b,α	46.27 b,α	69.91 b,α	58.76 a,β
2023	44.54 ab,α	42.18 a,α	281.36 a,α	463.70 c,β	506.37 a,α	798.38 c,β	44.38 ab,α	39.03 a,α	74.86 c,α	55.13 a,β

Abbreviations: MeJA: methyl jasmonate; GPE: grape pomace extract; IMC: index of cellular maturity; SMI: seed maturity index. Different letters in the same column (a–d) indicate significant differences according to the LSD test (*p* < 0.05) for the two studied factors (treatment and year) separately. For each extractability parameter, different letters in the same row (α, β) indicate significant differences between harvest times (*p* < 0.05).

**Table 4:** Color parameters in wines at the end of alcoholic fermentation.

	Treatment						Year			
	Control		MeJA		GPE		2022		2023	
	21 °Brix	23 °Brix	21 °Brix	23 °Brix	21 °Brix	23 °Brix	21 °Brix	23 °Brix	21 °Brix	23 °Brix
Anthocyanins **	353.08 ab,α	352.75 ab,α	340.25 ab,α	401.58 b,α	325.83 a,α	308.00 a,α	310.22 a,α	365.0 ab	369.22 b,α	343.22 ab,α
IC	6.856 ab,α	9.456 c,β	6.922 ab,α	9.445 c,β	6.181 a,α	7.979 b,β	6.499 a,α	8.971 b,β	6.813 a,α	9.130 b,β
Tint	0.495 a,α	0.570 c,β	0.522 ab,α	0.552 bc,α	0.503 a,α	0.534 abc,α	0.495 a,α	0.531 b,β	0.519 ab,α	0.574 c,β
L*	26.26 bc,α	18.75 a,β	24.75 b,α	19.08 a,β	28.97 c,α	22.88 ab,β	27.64 b,α	21.17 a,β	25.68 b,α	19.30 a,β
a*	58.47 cd,α	51.14 a,β	55.27 bc,α	51.46 ab,α	60.05 d,α	55.55 c,β	59.48 c,α	54.21 ab,β	56.39 bc,α	51.23 a,β
b*	34.21 ab,α	31.35 a,α	31.75 a,α	31.27 a,α	32.91 a,α	36.17 b,β	34.05 a,α	34.06 a,α	31.86 a,α	31.80 a,α
C*	67.77 bc,α	59.99 a,β	63.76 ab,α	60.24 a,α	68.55 c,α	66.29 bc,α	68.56 c,α	64.03 ab,β	64.83 bc,α	60.32 a,β
H*	30.35 ab,α	31.48 bc,α	29.85 ab,α	31.17 bc,α	28.75 a,α	33.06 c,β	29.79 a,α	32.10 b,β	29.51 a,α	31.72 b,β
Tannins **	667.72 a,α	968.47 b,β	678.00 a,α	1002.16 b,β	646.20 a,α	926.38 b,β	483.26 a,α	955.69 c,β	844.68 b,α	975.65 c,β
TP	32.60 a,α	41.45 b,β	32.35 a,α	42.08 b,β	30.26 a,α	38.20 b,β	33.06 b,α	45.47 d,β	30.41a,α	35.68 c,β

Abbreviations: MeJA: methyl jasmonate, GPE: grape pomace extract. ** expressed as mg/L. Different letters in the same row (a–d) indicate significant differences according to the LSD test (*p* < 0.05) for the two studied factors (treatment and year) separately. For each color wine parameter, different letters in the same row (α, β) indicate significant differences between harvest times (*p* < 0.05).

**Table 5:** Anthocyanin composition in grapes at harvest time.

	Treatment						Year			
	Control		MeJA		GPE		2022		2023	
	21 °Brix	23 °Brix	21 °Brix	23 °Brix	21 °Brix	23 °Brix	21 °Brix	23 °Brix	21 °Brix	23 °Brix
Df-3g*	62.37 ab,α	68.15 b,α	69.05 b,α	97.72 c,β	47.69 a,α	60.13 ab,α	68.56 ab,α	80.86 b,α	58.85 a,α	69.81 b,β
Cy-3g	50.21 a,α	120.34 b,β	72.25 a,α	141.50 b,β	39.73 a,α	74.25 a,α	74.82 b,α	130.32 c,β	33.30 a,α	93.71 b,β
Pet-3g	74.92 a,α	79.56 a,α	75.49 a,α	106.81 b,β	60.94 a,α	73.38 a,α	80.75 b,α	93.69 b,α	60.14 a,α	79.47 b,β
Pn-3g	49.17 a,α	130.22 c,β	53.92 ab,α	140.91 c,β	43.95 a,α	87.25 b,β	66.38 b,α	125.37 c,β	31.65 a,α	116.88 c,β
Mv-3g	208.81 a,α	230.34 a,α	190.24 a,α	297.32 b,β	193.36 a, α	234.09 a,α	230.12 b, α	267.74 b,α	164.82 a,α	240.08 b,β
Df-ac3g	6.15 a,α	5.84 a,α	6.41 a,α	6.66 a,α	5.26 a,α	5.62 a,α	4.18 a,α	4.62 a,α	7.69 b,α	7.46 b,α
Pet-ac3g	8.20 a,α	7.50 a,α	8.21 a,α	8.64 a,α	7.52 a,α	7.83 a,α	7.12 a,α	7.26 a,α	8.83 b,α	8.71 b,α
Pn-ac3g	5.67 a,α	8.63 b,β	5.92 a,α	8.75 b,β	5.51 a,α	6.64 ab,α	4.01 a,α	7.01 b,β	7.39 b,α	9.01 c,β
Mv-ac3g	26.31 ab,α	27.44 ab,α	24.08 a,α	31.38 c,β	26.67 ab,α	29.67 bc,α	26.86 ab,α	29.68 b,α	24.51 a,α	29.32 b,β
Pn-cum3g	14.24 ab,α	22.32 b,α	11.74 a,α	15.80 ab,α	13.50 ab,α	17.84 a,α	14.72 b,α	28.51 c,β	11.60 b,α	7.18 a,β
Mv-cum3g	52.06 a,α	43.43 a,α	46.33 a,α	39.27 a,α	51.89 a,α	45.95 a,α	56.79 c,α	67.06 d,β	43.39 b,α	28.71 a,β
Total *	558.12 a,α	743.80 bc,β	563.65 a, α	899.98 c,β	496.06 a,α	640.24 ab,α	634.35 b,α	842.15 c,β	444.20 a,α	680.40 b,β

Abbreviations: MeJA, methyl jasmonate; GPE, grape pomace extract; Df-3g, delphinidin-3-O-glucoside; Cy-3g, cyanidin-3-O-glucoside; Pet-3g, petunidin-3-O-glucoside; Pn-3g, peonidin-3-O-glucoside; Mv-3g, malvidin-3-O-glucoside; ac3g, acetyl-3-O-glucosides; cum3g, coumaryl-3-O-glucosides. * individual and total anthocyanins expressed as mg/kg grapes. Different letters in the same row (a–d) indicate significant differences according to the LSD test (*p* < 0.05) for the two studied factors (treatment and year) separately. For each anthocyanin compound, different letters in the same row (α, β) indicate significant differences between harvest times (*p* < 0.05).

**Table 6:** Anthocyanin composition in wines at the end of alcoholic fermentation.

	Treatment						Year			
	Control		MeJA		GPE		2022		2023	
	21 °Brix	23 °Brix	21 °Brix	23 °Brix	21 °Brix	23 °Brix	21 °Brix	23 °Brix	21 °Brix	23 °Brix
Df-3g*	22.49 b,α	20.12 ab,α	20.24 ab,α	21.58 ab,α	19.79 ab,α	18.51 a,α	21.09 a,α	19.28 a,α	20.59 a,α	20.86 a,α
Cy-3g	11.00 a,α	13.47 a,α	11.77 a,α	13.83 a,α	10.93 a,α	12.29 a,α	9.14 a,α	11.13 b,β	13.33 c,α	15.26 d,β
Pet-3g	34.22 a,α	32.35 a,α	29.60 a,α	32.16 a,α	31.12 a,α	28.85 a,α	34.56 b,α	32.50 ab,α	28.74 a,α	29.74 a,α
Cy-3g VitA	6.76 a,α	6.97 a,α	6.77 a,α	6.86 a,α	4.87 a,α	6.71 a,α	2.06 a,α	2.11 a,α	10.21 b,α	11.58 b,α
Pn-3g	17.97 a,α	30.90 c,β	17.60 a,α	27.42 c,β	19.47 a,α	23.61 b,β	18.99 a,α	26.23 b,β	17.71 a,α	28.38 b,β
Mv-3g	128.52 a,α	130.30 a,α	113.78 a,α	128.24 a,α	122.52 a,α	112.93 a,α	134.09 c,α	131.14 bc,α	109.13 a,α	116.51 ab,α
Pn-3g pyruvate	0.00 a,α	0.98 b,β	0.00 a,α	0.95 b,β	0.00 a,α	0.00 a,α	0.00 a,α	1.29 b,β	0.00 a,α	0.00 a,α
Df-ac3g	7.75 a,α	7.21 a,α	7.31 a,α	7.05 a,α	7.18 a,α	7.01 a,α	2.72 a,α	2.58 a,α	12.31 c,α	11.59 b,β
Mv-3g pyr(Vit A)	7.51 a,α	8.20 a,α	7.32 a,α	7.91 a,α	7.35 a,α	7.72 a,α	2.65 a,α	3.64 b,β	12.14 c,α	12.25 c,α
Mv-3g acet (Vit B)	7.25 a,α	7.43 a,α	7.42 a,α	7.80 a,α	7.38 a,α	7.48 a,α	2.75 a,α	3.14 b,β	11.95 c,α	12.00 c,α
Acetil VitA	0.00 a,α	0.98 b,β	0.00 a,α	0.00 a,α	0.00 a,α	0.00 a,α	0.00 a,α	0.65 b,β	0.00 a,α	0.00 a,α
Acetil VitB	5.71 b,α	0.00 a,β	3.82 b,α	0.94 a,β	0.00 a,α	0.96 a,α	0.00 a,α	1.27 a,α	6.35 b,α	0.00 a,α
Pet-ac-3g	8.63 a,α	7.75 a,α	7.87 a,α	7.73 a,α	7.83 a,α	7.71 a,α	3.62 a,α	3.50 a,α	12.60 c,α	11.96 b,β
Mv-3Og-ethyl epi2	0.99 a,α	0.98 a,α	0.98 a,α	0.96 a,α	0.95 a,α	0.99 a,α	1.95 b,α	1.92 b,α	0.00 a,α	0.00 a,α
Mv-3Og-ethyl epi3	2.92 a,α	4.93 a,α	4.79 a,α	4.87 a,α	4.80 a,α	6.76 a,α	1.99 a,α	2.14 a,α	6.35 b,α	8.89 b,α
Pn-ac3g	7.24 a,α	7.63 a,α	7.16 a,α	7.49 a,α	7.21 a,α	7.39 a,α	2.60 a,α	3.03 b,β	11.81 c,α	11.98 c,α
Mv-ac3g	20.10 b,α	16.50 ab,α	16.78 ab,α	15.46 a,α	17.00 ab,α	16.11 a,α	15.23 ab,α	14.52 a,α	20.70 c,α	17.53 b,β
Mv6cum-3g pyruvate	0.00 a,α	2.91 ab,α	5.71 b,α	0.00 a,β	3.81 ab,α	6.067 b,α	0.00 a,α	1.30 ab,α	6.35 c,α	5.08 bc,α
Mv6cum-3g acetaldehyde	7.28 a,α	8.34 a,α	6.77 a,α	7.49 a,α	7.16 a,α	7.70 a,α	2.51 a,α	3.59 b,β	11.74 c,α	12.09 c,α
Mv-3Og-ethyl epi4	7.26 a,α	7.20 a,α	6.93 a,α	6.94 a,α	7.15 a,α	7.10 a,α	2.54 a,α	2.56 a,α	11.69 b,α	11.60 b,α
Pn-cum3g	8.31 a,α	9.97 a,α	7.54 a,α	8.95 a,α	8.26 a,α	9.08 a,α	3.84 a,α	5.17 b,β	12.23 c,α	13.49 d,β
Mv-cum3g	23.85 c,α	24.12 c,α	17.47 a,α	20.01 ab,α	22.19 bc,α	22.30 bc,α	20.95 a,α	22.41 a,α	21.39 a,α	21.88 a,α
Total *	335.78 a,α	349.24 a,α	307.91 a,α	334.70 a,α	317.04 a,α	317.94 a,α	283.32 a,α	295.19 a,α	357.18 b,α	372.74 b,α

Abbreviations: MeJA, methyl jasmonate; GPE, grape pomace extract; Df-3g, delphinidin-3-O-glucoside; Cy-3g, cyanidin-3-O-glucoside; Pet-3g, petunidin-3-O-glucoside; Pn-3g, peonidin-3-O-glucoside; Mv-3g, malvidin-3-O-glucoside; ac3g, acetyl-3-O-glucosides; cum3g, coumaryl-3-O-glucosides; pyr, pyruvate; Vit, vitisin; epi, epicatechin. * individual and total anthocyanins expressed as mg/L. Different letters in the same row (a–d) indicate significant differences according to the LSD test (*p* < 0.05) for the two studied factors (treatment and year) separately. For each anthocyanin compound, different letters in the same row (α, β) indicate significant differences between harvest times (*p* < 0.05).

**Table 7:** Flavonol composition in grapes at harvest time.

	Treatment						Year			
	Control		MeJA		GPE		2022		2023	
	21 °Brix	23 °Brix	21 °Brix	23 °Brix	21 °Brix	23 °Brix	21 °Brix	23 °Brix	21 °Brix	23 °Brix
Myr-3-gU*	1.20 a,α	1.52 ab,α	0.67 a,α	2.49 b,β	0.73 a,α	1.51 ab,β	0.81 a,α	0.78 a,α	0.93 a,α	2.90 b,β
Myr-3gal	1.21 ab,α	2.41 c,β	0.73 a,α	2.06 c,β	0.89 a,α	1.94 bc,β	1.03 ab,α	1.45 b,α	0.86 a,α	2.83 c,β
Myr-3g	9.72 bc,α	15.94 d,β	6.30 a,α	12.54 cd,β	7.94 ab,α	12.14 c,β	8.24 a,α	14.01 b,β	7.74 a,α	13.07 b,β
Quer-3gal	1.09 a,α	3.67 a,α	1.67 a,α	2.86 a,α	1.44 a,α	1.74 a,α	2.81 b,α	5.43 c,β	0 a,α	0.08 a,α
Quer-3gU	11.95 d,α	9.36 bc,β	10.84 cd,α	8.79 ab,β	9.12 bc,α	7.06 a,β	10.17 bc,α	8.26 a,β	11.10 c,α	8.55 ab,β
Quer-3g	22.64 a,α	38.81 b,β	15.12 a,α	20.95 a,α	17.48 a,α	20.71 a,α	21.21 a,α	32.20 b,β	15.6 a,α	21.45 a,α
Lar-3g	1.69 a,α	2.98 b,β	1.01 a,α	2.57 b,β	1.50 a,α	2.47 b,β	1.51 a,α	2.48 b,β	1.29 a,α	2.87 b,β
Kam-3g	1.65 a,α	3.37 a,α	1.70 a,α	2.23 a,α	2.18 a,α	1.69 a,α	3.25 b,α	4.47 c,β	0.44 a,α	0.38 a,α
Iso-3g	0.12 a,α	0.01 a,α	0.01 a,α	0.01 a,α	0.01 a,α	0.01 a,α	0.01 a,α	0.01 a,α	0.01 a,α	0.01 a,α
Syr-3-g	0.64 a,α	1.22 b,β	0.48 a,α	1.08 b,β	0.64 a,α	1.03 b,β	0.65 a,α	1.16 b,β	0.52 a,α	1.07 b,β
Total *	51.94 ab,α	79.29 c,β	38.55 a,α	55.61 b,β	41.95 ab,α	50.31 ab,α	49.70 a,α	70.28 b,β	38.59 a,α	53.21 a,α

Abbreviations: MeJA, methyl jasmonate; GPE, grape pomace extract; Myr, myricetin; Quer, quercetin; Lar, laricitrin; Kam, kampherol; Iso, isorhamnetin; Syr, syrigetin, gU, O-glucuronide; gal, O-galactoside; g, O-glucoside. * individual and total flavonols expressed as mg/kg grapes. Different letters in the same row (a–d) indicate significant differences according to the LSD test (*p* < 0.05) for the two studied factors (treatment and year) separately. For each flavonol compound, different letters in the same row (α, β) indicate significant differences between harvest times (*p* < 0.05).

**Table 8:** Flavonol composition in wines at the end of alcoholic fermentation.

	Treatment						Year			
	Control		MeJA		GPE		2022		2023	
	21 °Brix	23 °Brix	21 °Brix	23 °Brix	21 °Brix	23 °Brix	21 °Brix	23 °Brix	21 °Brix	23 °Brix
Myr-3-gU*	1.67 a,α	2.38 ab,α	0.99 a,α	4.31 b,β	0.96 a,α	3.08 ab,α	0.85 a,α	0.95 a,α	1.57 a,α	5.56 b,β
Myr-3-gal	1.11 a,α	3.12 b,β	0.90 a,α	3.11 b,β	0.91 a,α	2.95 b,β	0.48 a,α	0.70 a,α	1.47 b,α	5.42 c,β
Myr-3-g	11.87 ab,α	16.29 ab,α	8.19 a,α	17.33 b,β	9.06 ab,α	16.27 ab,α	6.22 a,α	8.06 a,α	13.19 b,α	25.20 c,β
Quer-3-gal	0.40 a,α	0.90 a,α	0.39 a,α	0.66 a,α	0.50 a,α	0.63 a,α	0.85 c,α	1.30 d,β	0.00 a,α	0.16 b,β
Quer-3-gU	14.06 a,α	12.81 a,α	12.31 a,α	12.33 a,α	9.93 a,α	10.29 a,α	5.30 a,α	6.62 a,α	18.91 b,α	16.94 b,α
Quer-3-g	24.28 a,α	30.02 a,α	15.84 a,α	24.96 a,α	13.31 a,α	24.03 a,α	9.33 a,α	9.18 a,α	26.28 b,α	43.49 c,β
Lar-3-g	1.99 abc,α	3.70 cd,α	1.37 a,α	3.87 d,β	1.80 ab,α	3.55 bcd,α	1.22 a,α	1.90 b,β	2.22 b,α	5.51 c,β
Kam-3-g	0.94 ab,α	0.70 a,α	1.09 b,α	0.85 ab,α	0.84 ab,α	0.73 a,α	1.16 b,α	0.79 a,β	0.75 a,α	0.73 a,α
Iso-3-g	0.99 a,α	1.65 b,β	0.78 a,α	1.28 ab,α	0.74 a,α	1.35 ab,α	0.59 a,α	0.85 b,β	1.09 c,α	2.01 d,β
Syr-3-g	0.84 a,α	1.71 b,β	0.66 a,α	1.57 b,β	0.84 a,α	1.50 b,β	0.66 a,α	1.14 c,β	0.90 b,α	2.04 d,β
Total *	58.87 a,α	76.57 b,β	43.03 a,α	72.18 b,β	39.57 a,α	66.40 ab,α	27.92 a,α	34.62 a,α	66.40 b,α	104.82 c,β

Abbreviations: MeJA, methyl jasmonate; GPE, grape pomace extract; Myr, myricetin; Quer, quercetin; Lar, laricitrin; Kam, kampherol; Iso, isorhamnetin; Syr, syrigetin, gU, O-glucuronide; O-gal, O-galactoside; O-g, O-glucoside. * individual and total flavonols expressed as mg/L. Different letters in the same row (a–d) indicate significant differences according to the LSD test (*p* < 0.05) for the two studied factors (treatment and year) separately. For each flavonol compound, different letters in the same row (α, β) indicate significant differences between harvest times (*p* < 0.05).

**Table 9:** Proanthocyanidins in grapes at harvest time.

	Treatment						Year			
	Control		MeJA		GPE		2022		2023	
	21 °Brix	23 °Brix	21 °Brix	23 °Brix	21 °Brix	23 °Brix	21 °Brix	23 °Brix	21 °Brix	23 °Brix
mDP	15.66 b,α	13.29 ab,α	14.24 ab,α	15.78 b,α	15.25 ab,α	12.89 a,α	17.21 c,α	15.04 b,β	12.89 a,α	12.92 a,α
% Gal	1.51 b,α	1.75 c,β	1.54 bc,α	0.95 a,β	1.42 b,α	1.50 b,α	1.50 ab,α	1.56 b,α	1.48 ab,α	1.24 a,α
EGC-Ext*	234.56 bc,α	135.14 a,β	237.35 c,α	237.4 c,α	220.34 bc,α	160.55 ab,α	272.91 b,α	195.75 a,β	188.59 a,α	159.65 a,α
Cat-Ext	12.54 a,α	8.77 a,β	13.63 b,α	10.50 ab,α	12.62 b,α	11.03 ab,α	32.60 a,α	31.71 a,α	48.70 b,α	35.08 a,β
Epi-Ext	604.94 c,α	392.33 a,β	596.49 c,α	537.87 bc,α	531.80 bc,α	456.72 bc,α	568.76 b,α	504.31 ab,α	586.74 b,α	420.3 a,β
Cat-T	39.57 ab,α	29.63 a,α	44.28 b,α	32.06 a,β	38.21 ab,α	38.50 ab,α	32.60 a,α	31.71 a,α	48.78 b,α	35.08 a,β
Epi-T	18.76 ab,α	13.54 a,α	19.85 ab,α	22.39 b,α	17.65 ab,α	13.41 a,α	19.97 a,α	18.63 a,α	17.53 a,α	14.26 a,α
ECG-Ext	20.28 cd,α	15.53 abc,α	20.56 d,α	11.79 a,β	17.48 bcd,α	15.21 ab,α	20.55 b,α	17.61 b,α	18.32 b.α	10.74 a,β
ECG-T	0.62 ab,α	0.29 ab,α	0.86 b,α	0.00 a,β	0.42 ab,α	0.72 ab,α	0.19 ab,α	1.08 c,β	0.63 bc,α	0.04 a,β
Totalµg/g skin	8427.21 b,α	5407.84 a,β	7531.04 b,α	5565.62 a,β	7395.41 b,α	5731.76 a,β	8284.88 b,α	6049.96 b,β	7285.02 b,α	5086.85 a,β
Total mg/Kg	606.61c,α	358.54 a,β	540.85 bc,α	486.52 b,α	496.70 bc,α	368.60 a,β	589.49 c,α	480.22 b,β	506.61 bc,α	328.89 a,β

Abbreviations: MeJA, methyl jasmonate; GPE, grape pomace extract; mDP, mean degree polymerization; Gal, galloylation; EGC, epigallocatechin, Cat, catechin; Epi, epicatechin; ECG, epicatechin gallate; ext: extension units; T, terminal units. * expressed as mg/L. Different letters in the same row (a–d) indicate significant differences according to the LSD test (*p* < 0.05) for the two studied factors (treatment and year) separately. For each tannin parameter, different letters in the same row (α, β) indicate significant differences between harvest times (*p* < 0.05).

**Table 10:** Proanthocyanidins in wines at the end of alcoholic fermentation.

	Treatment						Year			
	Control		MeJA		GPE		2022		2023	
	21 °Brix	23 °Brix	21 °Brix	23 °Brix	21 °Brix	23 °Brix	21 °Brix	23 °Brix	21 °Brix	23 °Brix
GPm	5.43 a,α	4.90 a,α	5.24 a,α	5.48 a,α	5.13 a,α	4.99 a,α	4.68 a,α	4.88 a,α	6.02 c,α	5.24 b,β
% Gal	1.28 a,α	2.25 a,α	1.50 a,α	1.50 a,α	1.48 a,α	2.15 a,α	2.17 c,α	2.75 d,β	0.66 a,α	1.18 b,β
EGC-Ext*	105.28 ab,α	83.02 a,α	122.41 b,α	117.52 b,α	86.47 a,α	96.10 ab,α	85.43 a,α	109.46 bc,β	124.0 c,α	88.30 ab,β
Cat-Ext	22.79 a,α	21.05 a,α	23.06 a,α	22.57 a,α	19.74 a,α	23.08 a,α	10.18 a,α	12.97 b,β	33.56 c,α	31.50 c,α
Epi-Ext	310.42 a,α	261.31 a,α	314.10 a,α	283.50 a,α	248.08 a,α	282.79 a,α	229.09 a,α	296.68 b,β	352.64 c,α	225.07 ab,β
Cat-T	72.17 a,α	63.61 a,α	76.22 a,α	68.43 a,α	66.07 a,α	69.94 a,α	64.61 b,α	79.12 c,β	78.37 c,α	55.53 a,β
Epi-T	23.43 a,α	30.00 b,β	23.09 a,α	29.82 b,β	18.57 a,α	30.48 b,β	23.02 a,α	28.64 b,β	20.26 a,α	31.57 a,β
ECG-Ext	7.56 a,α	14.39 a,α	8.82 a,α	10.80 a,α	7.72 a,α	14.05 a,α	12.98 b,α	19.45 c,β	3.87 a,α	6.71 a,α
ECG-T	2.21 ab,α	2.34 ab,α	2.08 ab,α	1.31 a,α	1.70 ab,α	3.02 b,α	1.85 ab,α	2.98 b,α	2.14 ab,α	1.47 a,α
Total mg/L	543.69 a,α	475.74 a,α	569.81 a,α	533.98 a,α	448.39 a,α	519.49 a,α	426.38 a,α	549.33 bc,β	614.87 c,α	470.15 ab,β

Abbreviations: MeJA, methyl jasmonate; GPE, grape pomace extract; mDP, mean degree polymerization; Gal, galloylation; EGC, epigallocatechin, Cat, catechin; Epi, epicatechin; ECG, epicatechin gallate; ext: extension units; T, terminal units. * expressed as µg/berry. Different letters in the same row (a–d) indicate significant differences according to the LSD test (*p* < 0.05) for the two studied factors (treatment and year) separately. For each tannin parameter, different letters in the same row (α, β) indicate significant differences between harvest times (*p* < 0.05).

## Data Availability

The data are contained within this article.

## Abbreviations

The following abbreviations are used in this manuscript:

MeJA	methyl jasmonate
JA	jasmonic acid
JAs	jasmonates
GPE	grape pomace extract
SIAM	Agricultural Information System of the Region of Murcia
TP	total phenol content
IMC	index of cellular maturity
SMI	seed maturity index
WA	total wine anthocyanins
CI	color intensity
mDP	mean degree polymerization
LSD	least significant difference test
Df-3g	delphinidin-3-O-glucoside
Cy-3g	cyanidin-3-O-glucoside
Pet-3g	petunidin-3-O-glucoside
Pn-3g	peonidin-3-O-glucoside
Mv-3g	malvidin-3-O-glucoside
ac3g	acetyl-3-O-glucosides
cum	coumaryl-3-O-glucosides
pyr	pyruvate
Vit	vitisin
epi	epicatechin
BTH	Benzothiadiazole
Myr	myricetin
Quer	quercetin
Lar	laricitrin
Kam	kampherol
Iso	isorhamnetin
Syr	syrigetin
gU	O-glucuronide
gal	O-galactoside
g	O-glucoside
PAs	proanthocyanidins
% Gal	% galloylation
EGC	epigallocatechin
Cat	catechin
ECG	epicatechin gallate
ext	extension units
T	terminal units
